# Suppression of pancreatic beta cell apoptosis by Danzhi Jiangtang capsule contributes to the attenuation of type 1 diabetes in rats

**DOI:** 10.1186/s12906-016-0993-4

**Published:** 2016-01-27

**Authors:** Shuguo Zheng, Mengqiu Zhao, Yuanjie Wu, Zheng Wang, Younan Ren

**Affiliations:** 1 Department of Pharmacology, Wannan Medical College, Wuhu, 241002 China; 2 Department of Basic Theory of Chinese Medicine, Anhui University of Chinese Medicine, Hefei, 230038 China; 3 Department of Orthopedics and Traumatology, The First Affiliated Hospital of Anhui University of Chinese Medicine, Hefei, 230038 China

**Keywords:** Danzhi Jiangtang capsule, Diabetes, Pancreatic beta cells, Apoptosis, Pancreatic duodenal homeobox-1

## Abstract

**Background:**

Danzhi Jiangtang Capsule (DJC), a Chinese medicinal formula, has been clinically used for treatment of diabetes for many years. Previous studies have demonstrated that DJC was able to improve pancreatic islet function in diabetes, but the underlying mechanisms remained unclear.

**Methods:**

Streptozotocin (STZ) induced type 1 diabetic rats were treated with DJC for 6 weeks. Fasting plasma insulin and fasting plasma glucose were determined at the end of experiment. Antioxidant status was evaluated by measuring total antioxidant capacity, superoxide dismutase activity and malondialdehyde content in plasma and pancreas. Paraffin sections of pancreas were subjected to H&E staining, TUNEL staining and immunohistochemical examination. Protein levels of Bcl-2, Bax and pancreatic duodenal homeobox-1 (PDX-1) were measured by western blot analysis. Activities of Caspase-3 and Caspase-9 were determined with commercially available kits.

**Results:**

Supplementation with DJC resulted in a significant amelioration of type 1 diabetes as manifested by reduced blood glucose, increased fasting plasma insulin and improved body weight gains. The atrophy and reduction of pancreatic islets were also alleviated in DJC supplemented groups. DJC markedly reduced pancreatic beta cell apoptosis, with Bax protein down-regulated and Bcl-2 protein up-regulated significantly. The activities of caspase-3 and caspase-9 in pancreas were decreased evidently by DJC treatment. DJC effectively ameliorated oxidative stress in type 1 diabetic rats, with the expression of PDX-1 protein increased markedly.

**Conclusions:**

DJC was capable of attenuating STZ induced type 1 diabetes in rats, which might be attributed to the suppression of pancreatic beta cell apoptosis. This study would provide further evidence for clinical use of DJC in the management of diabetes.

## Background

Diabetes, a group of metabolic disease, is characterized by chronic hyperglycemia due to defects in insulin secretion and/or insulin action. Several lines of evidence indicated that diverse risk factors for type 2 diabetes such as overweight, physical inactivity and abdominal obesity can induce insulin resistance, a condition in which the body cells fail to respond effectively to insulin. In this situation, pancreatic beta cells have to produce more insulin to overcome this insensitivity, resulting in a state of hyperglycemia and hyperinsulinemia [1]. When pancreatic beta cells fail to keep up with the increased needs for insulin, excess glucose builds up in the bloodstream, leading ultimately to type 2 diabetes and various complications. Clinical data have demonstrated that, along with the progression of diabetes, early stage hyperinsulinemia due to insulin resistance transferred gradually to late stage hypoinsulinemia secondary to progressive beta cell damage, while blood glucose level remained high in the whole process [2]. It is well established that hyperglycemia plays a critical role in the progressive damage of pancreatic beta cells in diabetes [3], suggesting that protection of beta cells from hyperglycemia induced damage might be an effective approach to the management of diabetes.

Several lines of evidence indicated that chronic hyperglycemia could exacerbate pancreatic islet dysfunction by inducing beta cell apoptosis or by decreasing the activity of beta-cell specific transcription factors which regulate insulin production, resulting in a decreased beta cell mass and progressively impaired insulin secretion [4, 5]. In vitro studies also indicated that exposure to high level of glucose induced significant beta cell apoptosis and impaired insulin secretion in human islets and INS-1 cells [6, 7]. Data from clinical studies demonstrated that diabetic patients had markedly increased beta cell apoptosis and reduced beta-cell mass [8]. Since compensatory islet formation and beta-cell proliferation remained unchanged in diabetes, it is likely that the main reason for the decrease in beta-cell mass is increased cell apoptosis [9]. These findings suggest that suppression of beta cell apoptosis might be a potential therapeutic target for the treatment of diabetes.

Danzhi Jiangtang Capsule (DJC), a Chinese medicinal formula consisting of cortex moutan (21.6 %), heterophylly falsestarwort root (27.1 %), unprocessed rehmannia root (21.6 %), oriental waterplantain rhizome (16.2 %), dodder seed (10.8 %) and leech (2.7 %), possesses the properties of supplementing Qi, nourishing Yin and activating blood circulation. DJC is clinically used for treatment of diabetes, which is called Xiaoke in traditional Chinese medicine. Previous studies have shown that DJC was able to lower blood glucose in patients with type 2 diabetes and experimental diabetic rats [10, 11]. It could also improve pancreatic beta cell function in elderly diabetic patients [12]. However, the mechanisms underlying these beneficial effects of DJC remained unclear. In the present study, we investigated the effect of DJC on pancreatic beta cell apoptosis in STZ induced type 1 diabetic rats and the possible mechanisms implicated.

## Methods

### Drug preparation

The six components of DJC were provided and identified by The First Affiliated Hospital of Anhui University of Chinese Medicine. All these herbs except leech were extracted with boiled water for three times, followed by concentration of the mixed extracts. After grinding into fine powder, leech was mixed with the above mentioned extracts. Then the mixture was dried, grinded and capsulized for clinical use, with each capsule containing 0.4 g extract prepared from 8 g herbal medicine. For use in this experiment, DJC powder was dissolved in distilled water and given to rats intragastrically.

### Animals and induction of diabetes

Sixty male Wistar rats aged 7 weeks (weight: 200–220 g) were obtained from the Experimental Animal Center of Nanjing Medical University and maintained in a temperature and humidity controlled room with a 12 h light–dark cycle. Rats were fed a standard diet with free access to drinking water. After adaptive feeding for 1 week, all rats except 10 randomly allocated to Control group were injected intraperitoneally with streptozotocin (STZ, 50 mg/kg, Sigma, MO, USA) to induce type 1 diabetes. Ten days later, random blood glucose (RBG) was determined, and rats with RBG levels ≥16.7 mmol/L were defined as successful diabetic rats [13]. This study was conducted in accordance with the Guide for the Care and Use of Laboratory Animals and approved by the Committee on the Care and Use of Laboratory Animals of Anhui University of Chinese Medicine.

### Animal grouping and drug dosing

Thirty-six diabetic rats were randomly allocated to 3 groups, namely diabetic group (Model), low dose of DJC group (DJCL, 0.63 g/kg · d^−1^) and high dose of DJC group (DJCH, 1.26 g/kg · d^−1^). The low dose of Danzhi Jiangtang capsule was extrapolated from human dose through normalization to body surface area (equivalent dose). Rats in DJCH and DJCL groups were administered intragastrically with DJC for 6 weeks, while those in Control and Model groups were given distilled water of the same volume. Doses of DJC were adjusted according to the changes in body weight monitored biweekly. RBG was measured biweekly by glucose oxidase method using a commercial glucometer (Sinocare Incorporation, Changsha China).

### Biochemical analysis

At the end of the experiment, rats were fasted overnight and anaesthetized by intraperitoneal injection of pentobarbital sodium (40 mg/kg body weight). Blood samples were collected into EDTA-coated tubes and plasma was separated by centrifugation at 3000 g, 4 °C for 10 min (Eppendorf Corporation, Hamburg, Germany). Fasting plasma glucose (FPG) was determined by glucose oxidase method (Nanjing Jiancheng Bioengineering Company, Nanjing China) and fasting plasma insulin (FINS) was measured by enzyme-linked immunosorbent assay (Hufeng Biotech, Shanghai, China). Plasma levels of Total antioxidant capacity (TAC) and superoxide dismutase (SOD) activity were evaluated by ferric reducing antioxidant power test and xanthine oxidase method, respectively [14]. Malondialdehyde (MDA) content in plasma was determined by thiobarbituric acid method (Nanjing Jiancheng Bioengineering Company, Nanjing, China). Pancreatic tissues were homogenized in phosphate buffered saline (PBS) and the levels of SOD activity and MDA content in the homogenates were determined as described above. Activities of Caspase-3 and Caspase-9 were determined with Colorimetric assay kit (Beyotime institute of Biotechnology, Haimen, China). Protein concentrations in pancreatic homogenates were measured by Bradford’s method (Beyotime institute of Biotechnology, Haimen, China). All biochemical analyses were performed with a microplate reader (Thermo Fisher Scientific Incorporation, MA, USA).

### Histopathological examination

Tissues from the same region of pancreas were fixed in formalin at room temperature overnight, followed by dehydration and embedding in paraffin. After deparaffinage and rehydration, sections of 5 μm (Leica RM2245, Leica Biosystems, Nussloch, Germany) were subjected to hematoxylin and eosin (H&E) staining. Samples were examined under an Olympus IX51 microscope (Olympus Corporation, Tokyo, Japan) by a pathologist.

### Evaluation of pancreatic beta cell apoptosis

Pancreatic beta cell apoptosis was determined by terminal deoxynucleotidyl transferase (TdT)-mediated dUTP nick end labeling (TUNEL) assay. Paraffin sections (5 μm) were incubated with proteinase K (20 μg/ml, Invitrogen, CA, USA) at 37 °C for 20 min. Endogenous peroxidase activity was blocked by incubation with 3 % hydrogen peroxide in methanol. After rinsing with PBS, sections were subjected to TUNEL staining according to the manufacturer’s instructions (Roche Biochemicals, Mannheim, Germany). The conjugated horseradish peroxidase was visualized with diaminobenzidine (DAB, Sigma, MO, USA), followed by counterstaining with hematoxylin. Six randomly selected islets of each section were analyzed under high magnification (×400) with Image-Pro Plus 6.0 (Media Cybernetics MD, USA) for TUNEL-positive (apoptotic) cells, with the results expressed as percentage of apoptotic cells over total cells. The apoptotic rate of each animal represents the mean of 6 randomly selected islets.

### Immunohistochemical analysis

Paraffin sections were incubated with 3 % hydrogen peroxide in methanol for 30 min to block endogenous peroxidase activity. After antigen retrieval with microwave heating and blocking with goat serum, sections were incubated with monoclonal anti-body against Bax and Bcl-2 (Santa Cruz, CA, USA) at 4 °C overnight. Then the sections were incubated with biotinylated secondary antibody (Zhongshan Golden Bridge., Beijing, China) for 30 min, followed by incubation with horseradish peroxidase-conjugated streptavidin for 30 min at room temperature. The peroxidase was visualized by incubation with DAB solution in dark for 10 min and then the sections were counterstained with hematoxylin. Six randomly selected islets of each section were captured under high magnification (×400) and analyzed with Image-Pro Plus 6.0 for mean optical density [15]. The expression level of each animal represents the mean of 6 randomly selected islets.

### Western blot analysis

Pancreatic levels of Bcl-2, Bax and pancreatic duodenal homeobox-1 (PDX-1) protein were evaluated by western blot analysis. Total proteins were extracted with RIPA lysis buffer (Beyotime institute of Biotechnology, Haimen, China) supplemented with 1 mmol/L phenylmethanesulfonyl fluoride (PMSF, Sigma, MO, USA), followed by determination of protein concentrations by bicinchoninic acid (BCA) method (Beyotime institute of Biotechnology, Haimen, China). Then protein samples were subjected to SDS-polyacrylamide gel electrophoresis (Bio-Rad, CA, USA) and transferred to nitrocellulose membrane (Millipore, MA, USA). After blocking with 5 % skimmed milk in TBS containing 0.2 % Tween-20 (TBST), the membrane was incubated overnight at 4 °C with primary antibodies against Bcl-2, Bax, PDX-1 (Santa Cruz, CA, USA) and β-actin (Beyotime institute of Biotechnology, Haimen, China), followed by incubation with horseradish peroxidase (HRP) conjugated secondary antibody (Beyotime institute of Biotechnology, Haimen, China) for 1 h at room temperature. Bound HRP was visualized with an enhanced chemiluminescence substrate (ECL, Beyotime institute of Biotechnology, Haimen, China) and protein bands were analyzed for integrated optical density (IOD) with Quantity One software (Bio-Rad, CA, USA). The levels of PDX-1, Bcl-2 and Bax were normalized to that of β-actin.

### Statistical analysis

Data were presented as mean ± SD. Comparisons among groups were performed by one-way ANOVA followed by the least significant difference (LSD) test. A value of *P* < 0.05 was considered statistically significant. All statistical analysis was performed with SPSS statistical software package, version 21.0 (SPSS Incorporation, IL, USA).

## Results

### General conditions

**Fig. 1:**
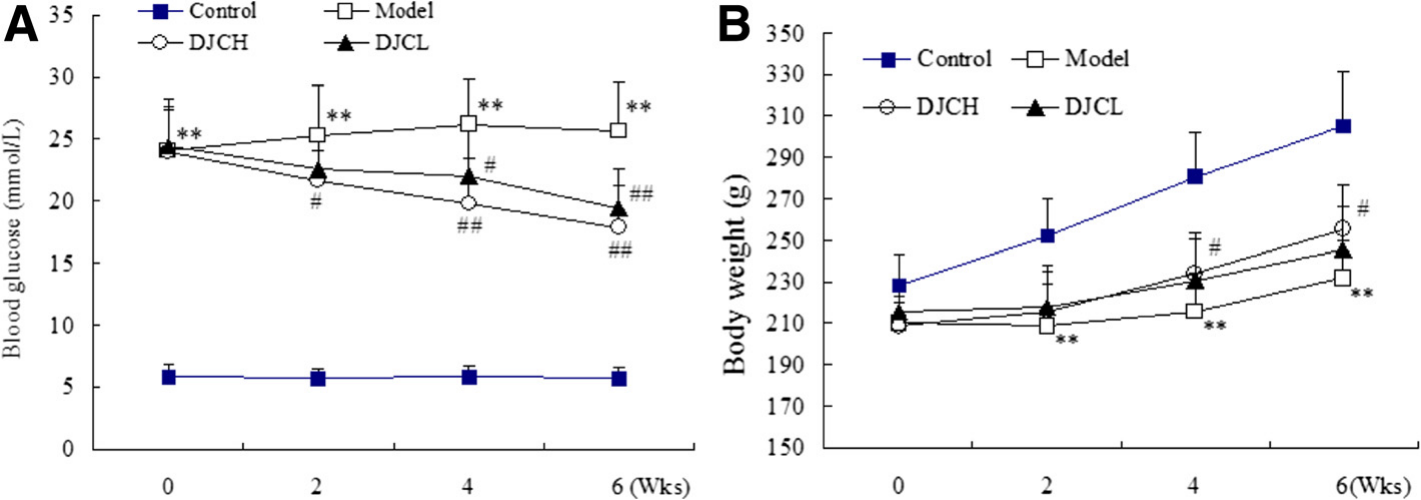
Effect of DJC on Blood Glucose **a** and Body Weight **b** in Rats. Data were presented as mean ± SD (*n* = 9 in Model group, *n* = 10 in the other groups). ^**^ *P* < 0.01 vs Control group; ^#^ *P* < 0.05, ^##^ *P* < 0.01 vs Model group

Thirty-six of 50 rats developed type 1 diabetes after intraperitoneal injection of STZ as evidenced by sustained hyperglycemia (≥16.7 mmol/L) 10 d after STZ injection. During the whole experiment, three of the 12 rats in Model group, two in DJCH group and two in DJCL group died of diverse causes including infection. As shown in Fig. 1, blood glucose in diabetic rats remained high all through the experiment (*P* < 0.01), while body weight gains were slowed down significantly as compared to that in Control group (*P* < 0.01). Supplementation with DJC resulted in a marked reduction in blood glucose (*P* < 0.05 or *P* < 0.01),and high dose of DJC significantly improved body weight gains in diabetic rats (*P* < 0.05). These results suggested that DJC was capable of ameliorating type 1 diabetes in rats.

### Effect of DJC on fasting plasma insulin and fasting plasma glucose

**Fig. 2:**
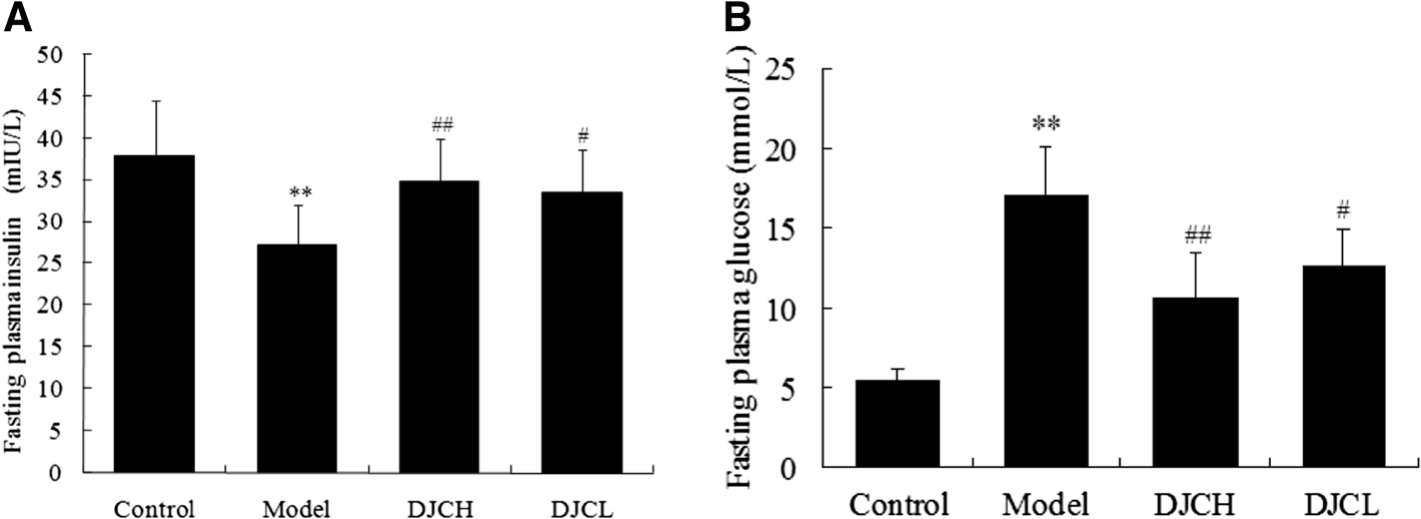
Effect of DJC on fasting plasma insulin **a** and fasting plasma glucose **b** in rats. Fasting plasma insulin and fasting blood glucose were determined by ELISA and glucose oxidase method, respectively. Data were presented as mean ± SD (*n* = 9 in Model group, *n* = 10 in the other groups). ^**^ *P* < 0.01 vs Control group; ^#^ *P* < 0.05, ^##^ *P* < 0.01 vs Model group

As shown in Fig. 2, FINS level in Model group was markedly lower than that in Control group (*P* < 0.01), while in DJC supplemented groups, levels of FINS were increased evidently (*P* < 0.05 or *P* < 0.01). Consistently, FPG in Model group was notably higher than that in Control group (*P* < 0.01), whereas supplementation with DJC resulted in a significant reduction in FPG in type 1 diabetic rats (*P* < 0.05 or *P* < 0.01). Regression analysis revealed that FPG correlated negatively with FINS (r = −0.576, *P* < 0.01). These results suggested that the beneficial effect of DJC on type 1 diabetes might be derived from the improvement of insulin secretion capacity.

### Effect of DJC on histopathological changes in pancreas

**Fig. 3:**
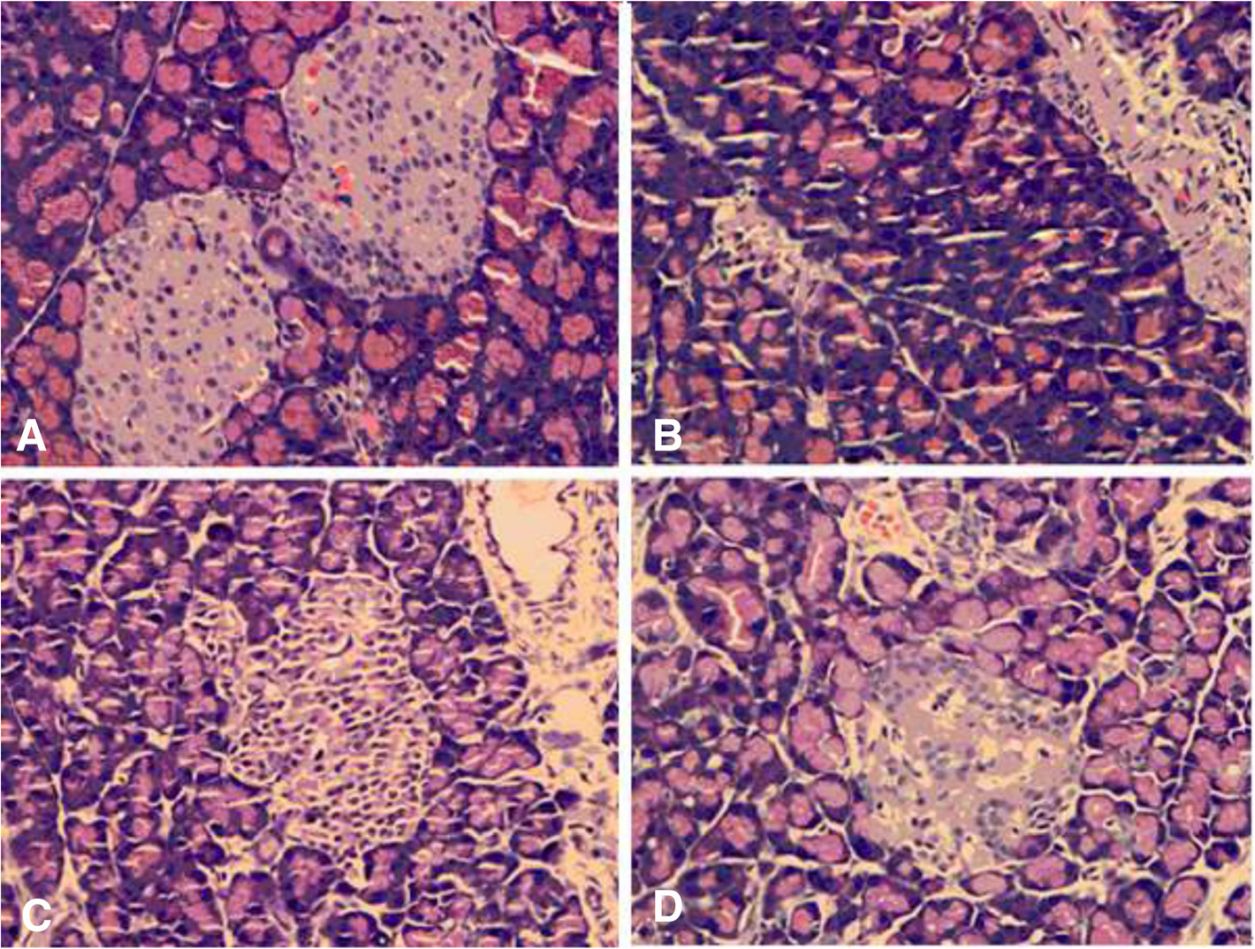
Representative microphotographs of histopathological examination of pancreas (×400). Paraffin sections of pancreas (5 μm) were stained with hematoxylin and eosin (H&E) and examined under a light microscope. **a**: Control group; **b**: Model group; **c**: DJCH group; **d**: DJCL group

As shown in Fig. 3, pancreas from Control group had quantitatively and morphologically normal islets, which were encapsulated in fibrous membranes and well demarcated from surrounding tissues. In model group, however, notably reduced and atrophied islets containing less beta cells were observed. In some islets, beta cells developed degranulation and vacuolar degeneration. As expected, treatment with DJC effectively alleviated the histopathological changes mentioned above, suggesting that the attenuation of type 1 diabetes by DJC might be derived from its protection of pancreatic islets.

### Effect of DJC on pancreatic beta cell apoptosis

**Fig. 4:**
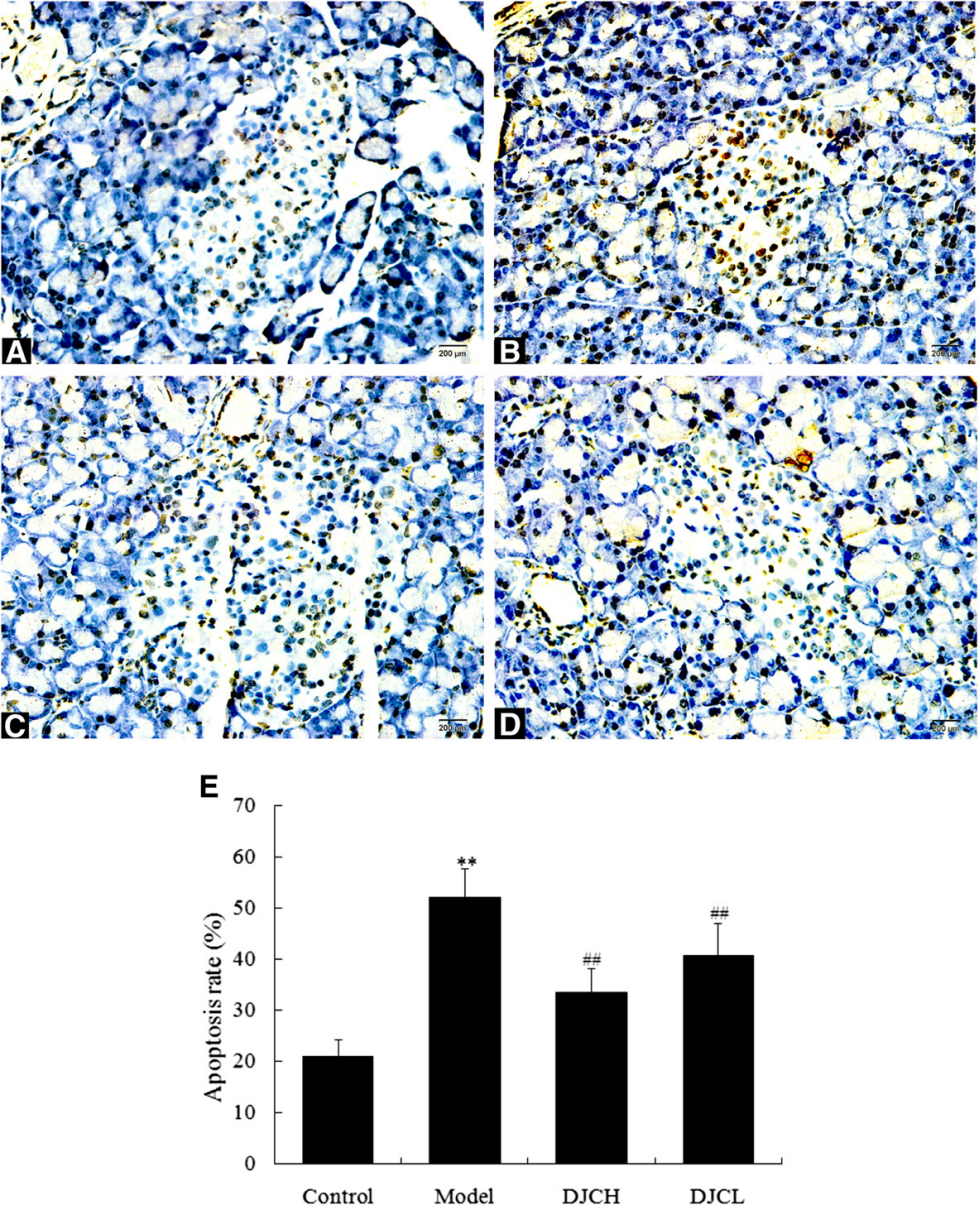
Effect of DJC on pancreatic beta cell apoptosis in rats. Paraffin sections of pancreas (5 μm) were subjected to TUNEL staining examination for beta cell apoptosis. Six randomly selected islets of each section were analyzed for apoptotic rate and averaged to serve as the apoptotic rate of each animal. Data were presented as mean ± SD (*n* = 9 in Model group, *n* = 10 in the other groups). **a**-**d**: Representative microphotographs of TUNEL staining (×400). **a**: Control group; **b**: Model group; **c**: DJCH group; **d**: DJCL group. **e**: Quantitative analysis of pancreatic islet cell apoptosis. ^**^ *P* < 0.01 vs Control group; ^##^ *P* < 0.01 vs Model group

Fig. 4 showed the results of TUNEL staining in pancreas. TUNEL positive cells in pancreatic islets increased notably in Model group (*P* < 0.01), while in DJC supplemented groups, TUNEL positive cells were reduced markedly (*P* < 0.01). These results indicated that DJC was capable of suppressing pancreatic beta cell apoptosis in type 1 diabetes, which might be responsible for its beneficial effect on diabetes.

### Effect of DJC on Bax and Bcl-2 protein expression

**Fig. 5:**
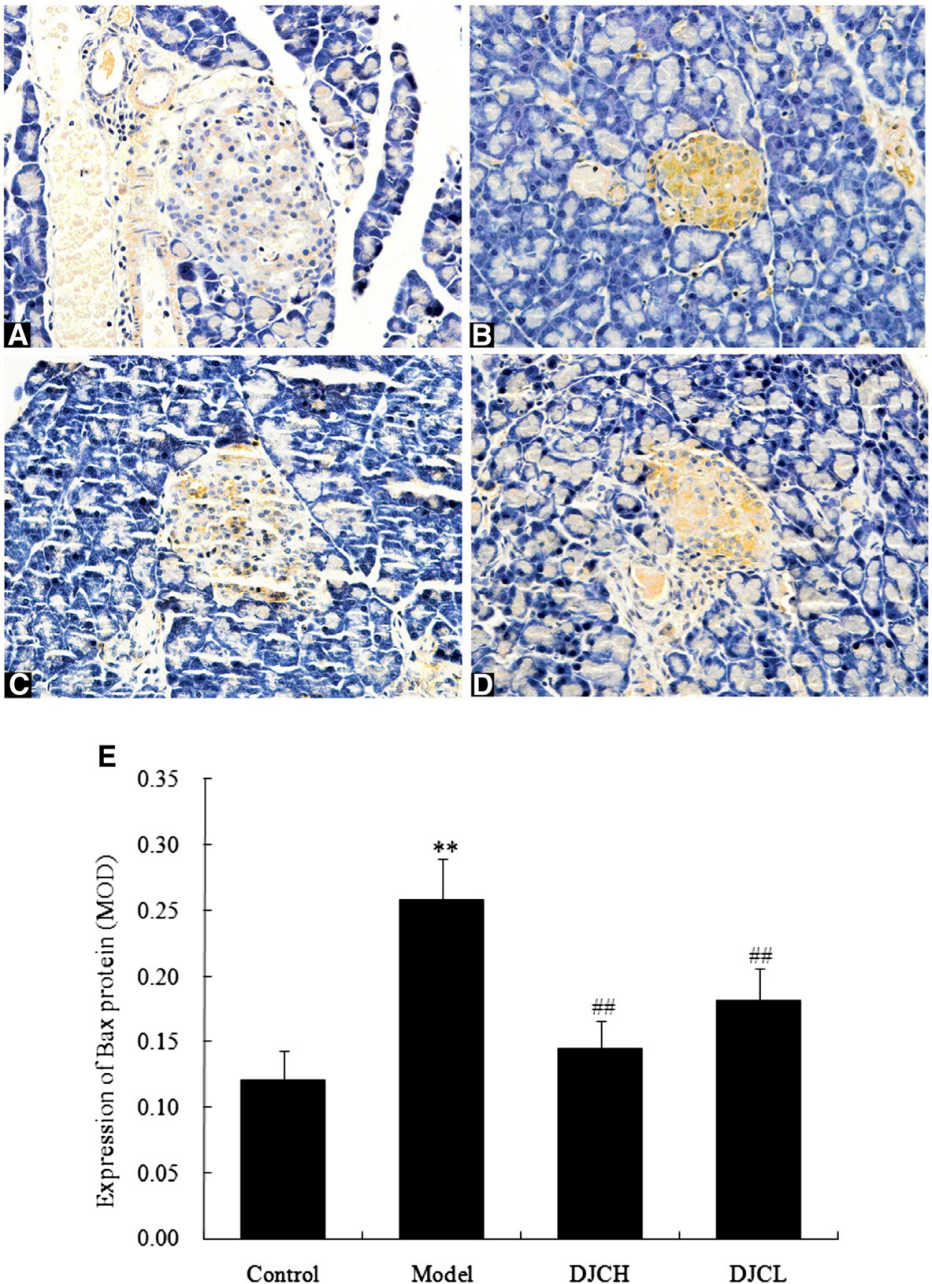
Effect of DJC on Bax protein level in rat pancreas. Six randomly selected islets of each section were analyzed for mean optical density and averaged to serve as the level of each animal. Data were presented as mean ± SD (*n* = 9 in Model group, *n* = 10 in the other groups). **a**-**d**: Representative microphotographs of immunohistochemical examination of Bax protein (×400). **a**: Control group; **b**: Model group; **c**: DJCH group; **d**: DJCL group. **e**: Quantitative analysis of Bax protein level. ^**^ *P* < 0.01 vs Control group; ^##^ *P* < 0.01 vs Model group

**Fig. 6:**
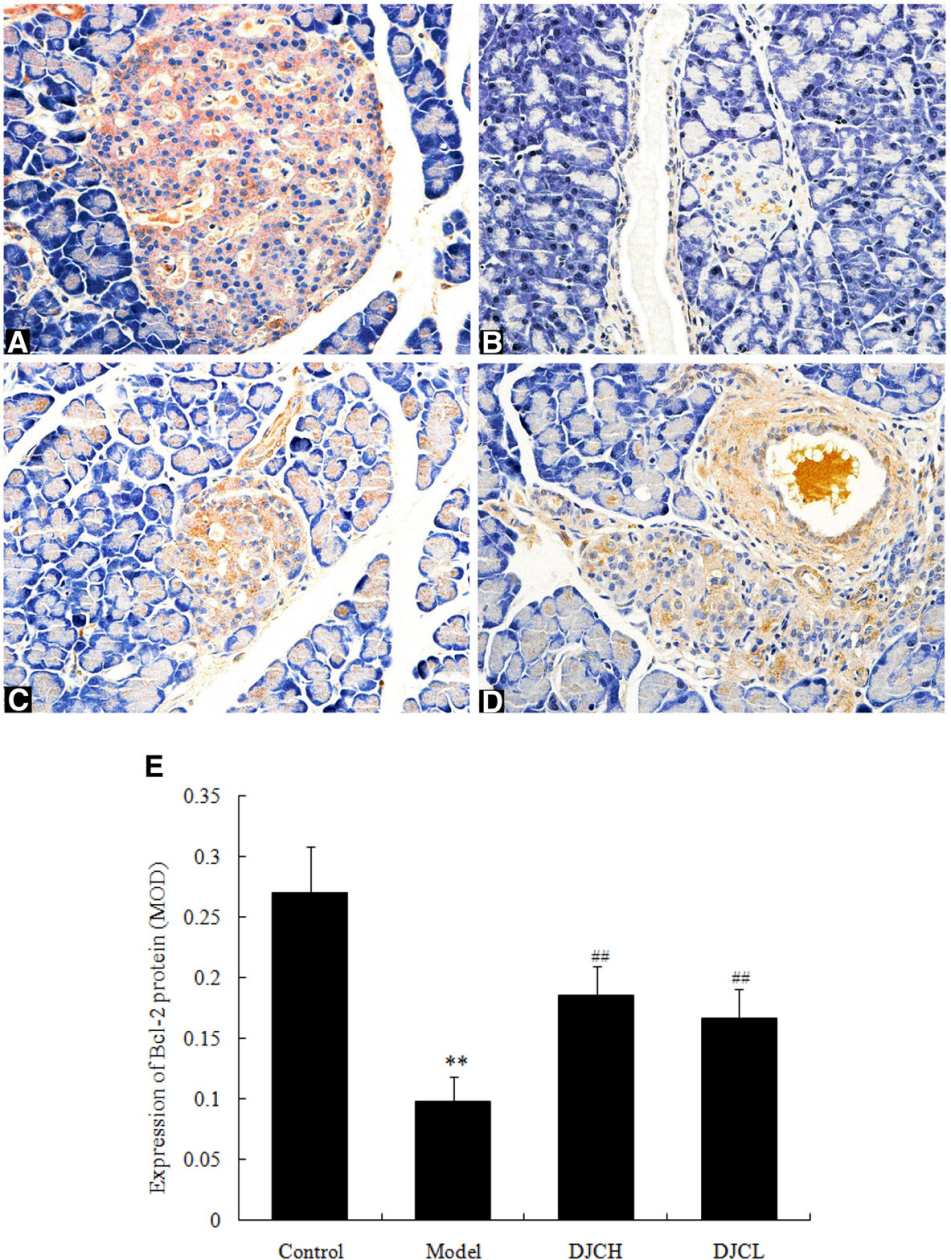
Effect of DJC on Bcl-2 protein level in rat pancreas. Six randomly selected islets of each section were analyzed for mean optical density and averaged to serve as the expression level of each animal. Data were presented as mean ± SD (*n* = 9 in Model group, *n* = 10 in the other groups). **a**-**d**: Representative microphotographs of immunohistochemical examination of Bcl-2 protein (×400). **a**: Control group; **b**: Model group; **c**: DJCH group; **d**: DJCL group. **e**: Quantitative analysis of Bcl-2 protein level. ^**^ *P* < 0.01 vs Control group; ^##^ *P* < 0.01 vs Model group

**Fig. 7:**
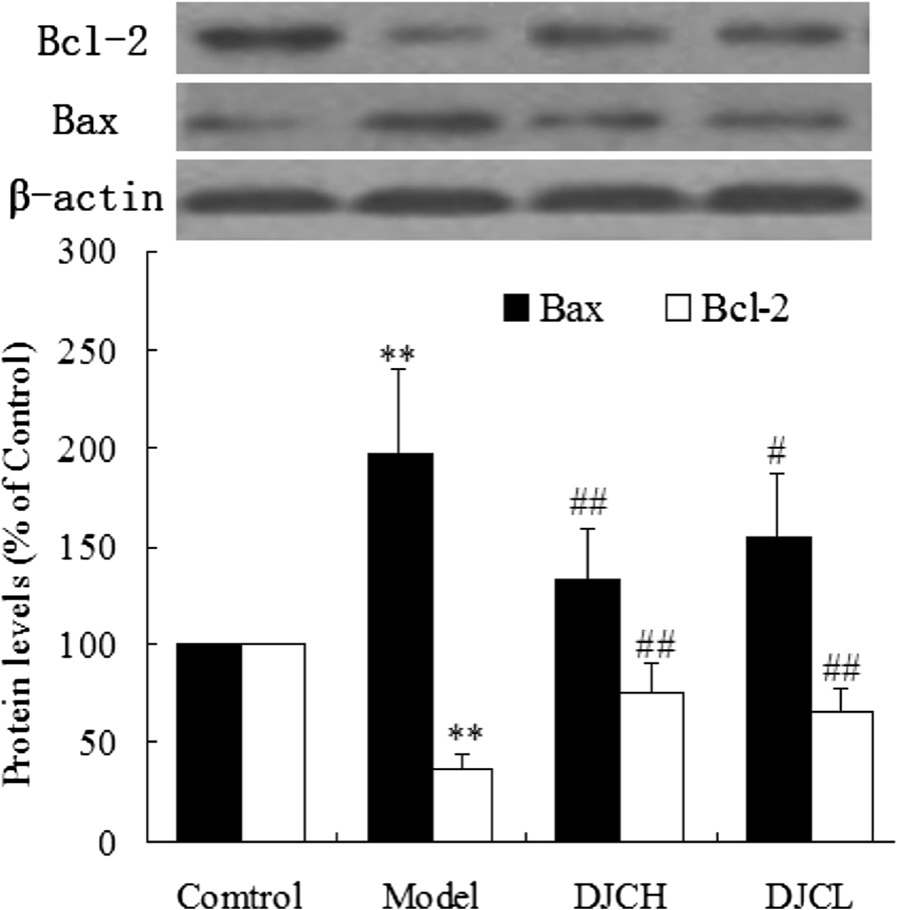
Western blot analysis of pancreatic levels of Bax and Bcl-2. Total protein were extracted from pancreas and subjected to western blot analysis. Levels of Bax and Bcl-2 protein were normalized to that of β-actin. Data were presented as percentage of that in Control group (Mean ± SD, *n* = 5). ^**^ *P* < 0.01 vs Control group; ^##^ *P* < 0.01 vs Model group

As shown in Figs. 5 and 6, significantly increased levels of Bax protein and decreased levels of Bcl-2 protein were observed in pancreatic islets from Model group (*P* < 0.01), while supplementation with DJC resulted in an evident down-regulation of Bax protein and up-regulation of Bcl-2 protein (*P* < 0.01). A similar result was also observed by Western blot analysis (Fig. 7). These findings were consistent with the suppressive effect of DJC on beta cell apoptosis, suggesting that the anti-apoptotic effect of DJC might be associated with its regulation of Bcl-2 family protein expression.

### Effect of DJC on the activities of Caspase-3 and Caspase-9

**Fig. 8:**
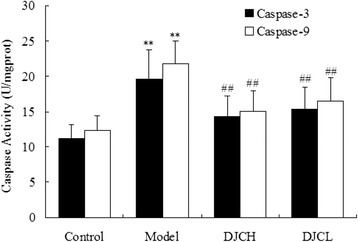
Effect of DJC on the activities of Caspase-3 and Caspase-9. The activities of caspase-3 and Caspase-9 in pancreas were determined by using Caspase colorimetric assay Kit. Data were presented as mean ± SD (*n* = 9 in Model group, *n* = 10 in the other groups). ^**^ *P* < 0.01 vs Control group; ^##^ *P* < 0.01 vs Model group

As shown in Fig. 8, the activities of Caspase-3 and Caspase-9 in pancreas from Model group were significantly higher than those from Control group (*P* < 0.01), while in DJC supplemented groups, the activities of both caspases were decreased evidently (*P* < 0.01). These results indicated that inhibition of Caspase-3 and Caspase-9 activation might be a potential mechanism underlying the anti-apoptotic effect of DJC in type 1 diabetic rats.

### Effect of DJC on PDX-1 protein expression

**Fig. 9:**
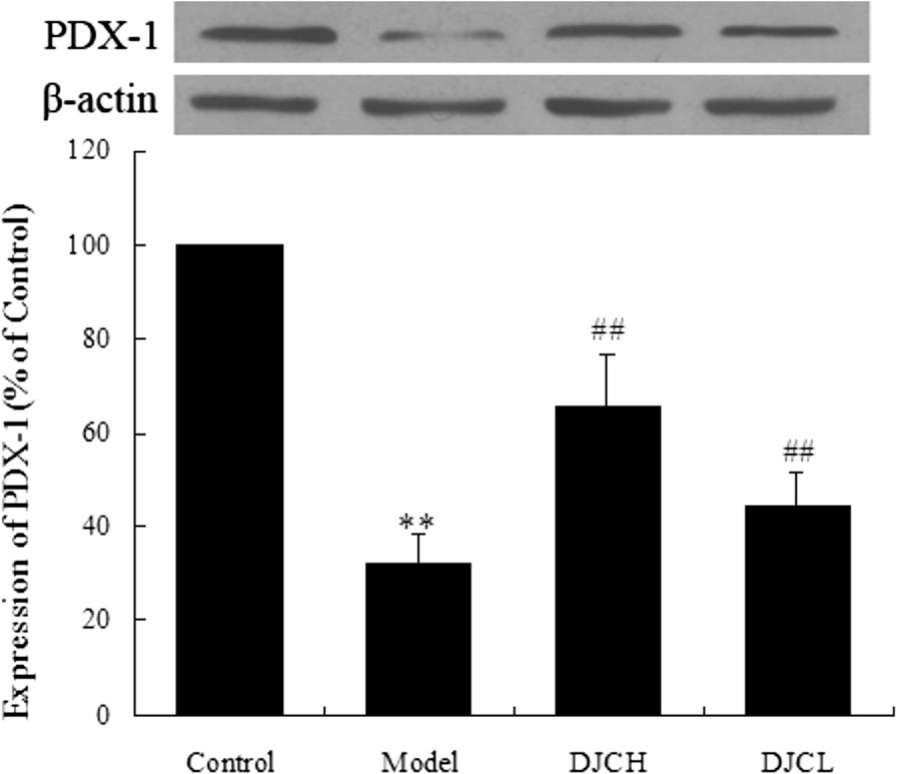
Effect of DJC on PDX-1 protein expression. Total protein extracted from pancreas were subjected to western blot analysis. Levels of PDX-1 protein were normalized to that of β-actin. Data were presented as percentage of that in Control group (Mean ± SD, *n* = 5). ^**^ *P* < 0.01 vs Control group; ^##^ *P* < 0.01 vs Model group

As shown in Fig. 9, level of PDX-1 protein in pancreas from Model group was markedly lower than that from Control group (*P* < 0.01), whereas in DJC supplemented groups, PDX-1 protein levels were increased markedly (*P* < 0.01).

### Effect of DJC on antioxidant status in rats

As shown in Table 1, plasma levels of TAC and SOD activity were significantly decreased in Model group (*P* < 0.01), while MDA level was increased markedly (*P* < 0.01). Consistently, a significantly decreased level of SOD activity and increased content of MDA were observed in pancreas from Model group (*P* < 0.01). These results suggested that diabetic rats developed significant oxidative stress. Supplementation with DJC notably enhanced TAC and SOD activity in both plasma and pancreatic tissues (*P* < 0.05 or *P* < 0.01), with the levels of MDA decreased markedly (*P* < 0.05 or *P* < 0.01). These results indicated that DJC was able to enhance antioxidant capacity and reduce oxidative damage in type 1 diabetic rats.

**Table 1:** Effect of DJC on antioxidant status in type 1 diabetic rats

Groups	Plasma TAC (U/mL)	Plasma SOD (U/mL)	Plasma MDA (nmol/mL)	Pancreatic SOD (U/mgprot)	Pancreatic MDA (nmol/mgprot)
Control	12.5 ± 1.8	241.4 ± 17.7	2.2 ± 0.4	10.4 ± 1.5	1.5 ± 0.3
Model	7.8 ± 1.2^**^	198.9 ± 21.8^**^	3.3 ± 0.5^**^	6.7 ± 1.3^**^	2.3 ± 0.4^**^
DJCH	10.6 ± 1.5^##^	230.7 ± 18.9^##^	2.3 ± 0.4^##^	9.2 ± 1.3^##^	1.7 ± 0.3^##^
DJCL	9.9 ± 1.5^#^	225.8 ± 14.5^##^	2.6 ± 0.4^#^	8.5 ± 1.6^#^	1.8 ± 0.3^#^

Data were presented as mean ± SD, *n* = 9 in Model group, *n* = 10 in the other groups)

^**^ *P* < 0.01 vs Control group; ^#^ *P* < 0.05, ^##^ *P* < 0.01 vs Model group

## Discussion

Increasing evidence indicated that chronic hyperglycemia can not only cause damage to various organs of the body, but also further exacerbate insulin deficiency by inducing pancreatic beta cell apoptosis [16], forming a vicious cycle leading to progressive deterioration of beta cell function [17]. In the present study, intraperitoneal injection of STZ induced severe hyperglycemia and reduced fasting plasma insulin levels in rats, accompanied by a significantly decrease in body weight gains. Consistently, histopathological examination showed that injection of STZ induced significant atrophy and reduction of pancreatic islets. These results suggested that rat model of type 1 diabetes was established successfully. Supplementation with DJC resulted in a marked increase of FINS and reduction of blood glucose, with the general conditions including body weight gains improved evidently. These results suggested that DJC was capable of attenuating STZ-induced type 1 diabetes in rats and the beneficial effects might be derived from protection of pancreatic islet function. This was in accordance with a previous clinical observation [12], but was inconsistent with another report that DJC decreased FINS in diabetic patients [18]. This discrepancy might be due to the reason that the patients recruited in the former study were all aged people, which might be at a later stage of diabetes with beta cell function seriously damaged. These findings suggested that DJC might have a bidirectional regulation effect on insulin secretion in diabetes, which was usually considered a main advantage of Chinese herbal medicine.

A large amount of evidence indicated that chronic hyperglycemia could induce oxidative stress and lead to an increased accumulation of reactive oxygen species (ROS), which can cause damage to various types of cells [19]. Owing to relatively lower expression level of antioxidant enzymes such as superoxide dismutase (SOD), pancreatic beta cells are more susceptible to ROS induced damage and apoptosis [20], which can lead to a progressively impaired insulin secretion capacity. Excessive ROS can induce beta cell damage through interference with intracellular signal transduction besides direct oxidative injury [21]. Pancreatic and duodenal homeobox-1 (PDX-1), a transcription factor expressed mainly in pancreatic beta cells, plays a critical role in pancreas development, beta cell maturation and survival, and the expression of a number of beta-cell specific genes including insulin [22]. ROS can reduce insulin synthesis and induce beta cell apoptosis through down-regulation of PDX-1 protein expression [23]. In the present study, diabetic rats developed significant oxidative stress as manifested by decreased levels of SOD activity and total antioxidant capacity (TAC) and increased content of MDA, which are usually used as biomakers of in vivo oxidative stress [24]. In line with previous reports, expression of PDX-1 protein was down-regulated significantly in type 1 diabetic rats, accompanied by a significantly decreased level of FINS and increased apoptotic rate of pancreatic beta cells. By contrast, supplementation with DJC resulted in a significant enhancement of antioxidant capacity and decrease of MDA content in diabetic rats, suggesting that DJC was able to ameliorate oxidative stress in type 1 diabetic rats. Consistent with this result, supplementation with DJC led to an evident up-regulation of PDX-1 protein, accompanied by a marked reduction of pancreatic beta cell apoptosis. These results indicated that the beneficial effect of DJC on diabetes might be derived from suppression of pancreatic beta cell apoptosis, which might be related to its alleviation of oxidative stress and up-regulation of PDX-1 protein expression.

In addition, immunohistochemical and western blot analysis all demonstrated that supplementation with DJC resulted in a significant up-regulation of Bcl-2 protein and down-regulation of Bax protein in pancreas. As a critical regulator of cell apoptosis, Bcl-2 family protein consists of both pro-apoptotic and anti-apoptotic members [25]. Among these proteins, Bax plays a pro-apoptotic role by inducing the release of cytochrome C from mitochondria, leading to caspase activation cascades and cell apoptosis. On the contrary, Bcl-2 plays an anti-apoptotic role either by sequestering pro-caspases or by preventing the release of cytochrome C [26]. In consistence with the regulatory effect of DJC on Bcl-2 related proteins, treatment with DJC markedly decreased the activities of Caspase-3 and Caspase-9. These results indicated that regulation of Bcl-2 family protein expression and subsequent inhibition of Caspases activation might also play a role in the anti-apoptotic effect of DJC in diabetes.

DJC consists of six Chinese herbs, most of which have been demonstrated to possess antioxidant activity [27–29]. As a compound formula, DJC has been shown to increase serum activities of SOD and GPx and decrease serum content of MDA in patients with diabetes [30]. Our previous studies also demonstrated that DJC was able to increase the activities of SOD and GPx in pancreatic tissues from diabetic rats [31, 32]. The findings of the present study were consistent with these reports, indicating that DJC can increase antioxidant capacity in diabetes through enhancement of antioxidant enzymes activity. In addition, DJC has been shown to decrease the expression of nicotinamide adenine dinucleotide phosphate (NADPH) oxidase subunit p22phox in diabetic rats [31]. As a main source of endogenous ROS in most mammalian cell types, NADPH oxidase can be activated by hyperglycemia, resulting in an increased production of ROS and oxidative damage. Previous studies have shown that suppression of NADPH oxidase activity or protein expression can markedly decrease hyperglycemia induced ROS production in pancreatic beta cells and improve insulin secretion function [33]. These findings suggested that, besides enhancement of antioxidant capacity, suppression of ROS production might also contribute to the antioxidant capacity and beneficial effect of DJC in diabetes.

## Conclusions

In conclusion, this study demonstrated that DJC was capable of attenuating STZ induced type 1 diabetes in rats, which might be attributed, at least partly, to the suppression of pancreatic beta cell apoptosis. These findings were in accordance with clinical observations and would provide further support for clinical use of DJC in the management of diabetes.

## Abbreviations

PDX-1: Pancreatic duodenal homeobox-1
RBG: Random blood glucose
FPG: Fasting plasma glucose
FINS: Fasting plasma insulin
TAC: Total antioxidant capacity
SOD: Superoxide dismutase
MDA: Malondialdehyde
TUNEL: Terminal deoxynucleotidyl transferase (TdT)-mediated dUTP nick end labeling
DAB: Diaminobenzidine
BCA: Bicinchoninic acid
HRP: Horseradish peroxidase
NADPH: Nicotinamide adenine dinucleotide phosphate
